# Supplementary material for: Nationwide health, socio-economic and genetic predictors of COVID-19 vaccination status in Finland
Source: Nat Hum Behav. 2023 Apr 20;7(7):1069–83. doi: 10.1038/s41562-023-01591-z (PMC10365990; doi:10.1038/s41562-023-01591-z)
Supplement: Supplementary file 1 — Supplementary Methods, references, legends for Supplementary Tables 1–9 and the full list of FinnGen consortium members. [file 41562_2023_1591_MOESM1_ESM.pdf]

nature human behaviour

Article https://doi.org/10.1038/s41562-023-01591-z

Nationwide health, socio-economic and genetic predictors of COVID-19 vaccination status in Finland

In the format provided by the authors and unedited

Table of contents

Table of contents **1**
Supplementary Methods **2**
Supplementary references **4**
Supplementary Tables **4**
FinnGen consortium full list of members **6**

Supplementary Methods

FinnGen

Genotyping and quality control

FinnGen consists of prospectively recruited samples and a series of legacy cohorts with genotypes already available. Prospective samples were genotyped using the ThermoFisher Axiom custom array which tags a total of 655,973 variants. Genotype calling was performed using the Array Power Tools software. Legacy cohorts were genotyped using various Illumina arrays and genotype calling was performed using either GenCall or zCall algorithms.

For both prospective and legacy cohorts the following quality control metrics were used.

Samples were removed if:

- Pihat was > 0.9 and the samples were not monozygotic or replicates
- There was a discrepancy between reported sex and genetically determined sex (F-value ≤ 0.3 for females and ≥ 0.8 for males)
- Missingness was ≥ 5%
- Heterozygosity was ±4 standard deviations from the population average
- Pihat was > 0.1 with 14 or more samples
- Samples were ±4 standard deviations away from the population average according to the first two genetic principal components.

Samples were tagged should there be evidence of a mendelian error or contain replicate samples with over 50,000 discrepancies.

Variants were removed if:

- The variant failed the Hardy-Weinberg Equilibrium test (p-value < 10^{-6})
- The variant had a call rate < 98%

Imputation

Pre-phasing was performed using Eagle 2.3.5 and samples were imputed using the SiSu v3 imputation reference panel. This reference panel is specific to the Finnish population, containing high-coverage (25-30x) whole-genome sequencing data from 3,775 Finns and 16,962,023 variants with minor allele count ≥ 3. After imputation, 16,387,711 variants were imputed with high quality (INFO > 0.6).

Ancestry assignment

Firstly, the FinnGen samples were combined with the 1000 genomes phase 3 dataset. Genetic principal components were calculated using a subset of 49,451 pruned SNPs.

Aberrant was used to identify and remove samples that deviated from the main cluster. A probability of belonging to either a North-Western European or Finnish population was calculated by firstly performing PCA with individuals belonging to these ancestries from 1000 genomes data. FinnGen samples were then projected onto this PCA space and Mahalanobis distances calculated for each sample against each of the two ancestries. Samples were retained if there was ≥ 95% probability of belonging to the Finnish ancestry cluster.

Estonian Biobank

Estonian Biobank (EstBB) is a population-based cohort with a rich variety of phenotypic and health-related information collected for each participant (Leitsalu et al. 2015). At recruitment, participants signed a consent allowing follow-up linkage of their electronic health records (EHR), thereby providing a longitudinal collection of their phenotypic information. The EstBB database includes health records from the national Health Insurance Fund Treatment Bills (from 2004), Tartu University Hospital (from 2008), and North Estonia Medical Center (from 2005), and data from different registries (causes of death, cancer, etc.). For all the participants EstBB provides information on the diagnoses in ICD-10 coding and information on drug dispensing data, including drug ATC codes, prescription status and purchase date (if available).

Genotyping and quality control

Genotyping of DNA samples from the Estonian Biobank was done at the Core Genotyping Lab of the Institute of Genomics, University of Tartu using the Illumina Global Screening Arrays (GSAv1.0, GSAv2.0, and GSAv2.0_EST). Altogether 206,448 samples were genotyped and then PLINK format files were created using Illumina GenomeStudio v2.0.4. During the quality control all individuals with call-rate < 95% or mismatching sex that was defined based on the heterozygosity of X chromosome and sex in the phenotype data, were excluded from the analysis. Variants were filtered by call-rate < 95% and HWE p-value < 1e-4 (autosomal variants only). Variant positions were updated to Genome Reference Consortium Human Build 37 and all variants were changed to be from TOP strand using reference information provided by Dr. Will Rayner from the University of Oxford (https://www.well.ox.ac.uk/~wrayner/strand/). After QC the dataset contained 202,910 samples for imputation.

Imputation

Before imputation variants with MAF<1% and Indels were removed. Prephasing was done using the Eagle v2.3 software (Loh et al. 2016) (number of conditioning haplotypes Eagle2 uses when phasing each sample was set to: --Kpbwt=20000) and imputation was carried out using Beagle v.18May20.d20 (S. R. Browning and Browning 2007; B. L. Browning, Zhou, and Browning 2018) with an effective population size ne=20,000. As a reference, Estonian population specific imputation reference of 2297 WGS samples was used (Mitt et al. 2017).

Ancestry assignment

Further, EstBB samples were combined with the 1000 genomes phase 3 dataset for ancestry analysis. Genetic principal components were calculated using a subset of quality

controlled and pruned genotyped SNPs. This was further used to identify and remove samples that deviated from the main cluster.

Supplementary references

Browning, Brian L., Ying Zhou, and Sharon R. Browning. 2018. “A One-Penny Imputed Genome from Next-Generation Reference Panels.” American Journal of Human Genetics 103 (3): 338–48. https://doi.org/10.1016/j.ajhg.2018.07.015.

Browning, Sharon R, and Brian L Browning. 2007. “Rapid and Accurate Haplotype Phasing and Missing-Data Inference for Whole-Genome Association Studies by Use of Localized Haplotype Clustering.” American Journal of Human Genetics 81 (5): 1084–97. https://doi.org/10.1086/521987.

Leitsalu, Liis, Helene Alavere, Mari-liis Tammesoo, Erkki Leego, and Andres Metspalu. 2015. “Linking a Population Biobank with National Health Registries — The Estonian Experience.” Journal of Personalized Medicine, 96–106. https://doi.org/10.3390/jpm5020096.

Loh, Po-Ru, Petr Danecek, Pier Francesco Palamara, Christian Fuchsberger, Yakir A Reshef, Hilary K Finucane, Sebastian Schoenherr, et al. 2016. “Reference-Based Phasing Using the Haplotype Reference Consortium Panel.” Nature Genetics 48 (11): 1443–48. https://doi.org/10.1038/ng.3679.

Supplementary Tables

Supplementary Tables are provided in a separate Excel file, here we list just the captions of the tables.

Supplementary Table 1. Predictive performance (measured using Area Under Receiver-operator characteristics curve, AUC of the manually curated predictor categories when predicting COVID-19 vaccination status. AUC is reported from XGBoost classifier models (except logistic regression was used for PRS and baseline in FinnGen, see Methods for details) that include all predictors from the corresponding predictor category of interest and age and sex as variables (see Methods for details). Baseline models (including only age and sex as predictors) were trained separately for the population-wide FinRegistry dataset, and the subset of the Finnish population genotyped in FinnGen. Percentage of improved over baseline was computed with respect to the baseline model of the corresponding study population.

Supplementary Table 2. Predictive performance (measured using Area Under Receiver-operator characteristics curve, AUC of the individual predictors when predicting COVID-19 vaccination status. AUC is reported from a Lasso classifier model that includes the predictor of interest and age and sex as variables (see Methods for details). If the predictor is not binary, isBinary column value is FALSE. N is the total number of individuals in the study population with the predictor, and N_among_vaccinated the same number among the vaccinated individuals. N_NA describes the number of study population individuals with missing value for the predictor.

Supplementary Table 3. Associations between COVID-19 vaccination status and each of the individual predictors. Odds ratios were computed adjusting for age and sex, as described in the Methods. P-values are two-sided and were calculated by dividing the coefficient values by their standard errors and observing the probability mass corresponding to more extreme values from both tails of the standard normal distribution (as in R package glm). Multiple hypothesis testing adjustment was computed using the Benjamini-Hochberg method. If the predictor is not binary, isBinary column value is FALSE (and N and N_among_vaccinated are missing). N is the total number of individuals in the study population with the predictor, and N_among_vaccinated the same number among the vaccinated individuals. N_NA describes the number of study population individuals with missing value for the predictor.

Supplementary Table 4. Hyperparameter spaces tested during training of the XGBoost models. Column names correspond to the XGBoost parameter names. Default values were used for parameters not listed here. Tested parameter ranges are given as Python expressions.

Supplementary Table 5. Pleiotropic associations of COVID vaccination lead variants. Discovered using Open Targets Genetics. P-values relating to the GWAS (FinnGen P, Estonia Biobank P and Meta-Analysis P) are two-sided and were calculated by dividing the coefficient values (Effect) by their standard errors and observing the probability mass corresponding to more extreme values from both tails of the standard normal distribution (as in R package glm). A bonferroni-corrected alpha of P-value < 5x10-8 was used as is standard for GWAS. The P-value for heterogeneity was two-sided and calculated from the Cochrans Q Test statistic which has a chi-square distribution and 1 degree of freedom. A bonferroni-corrected alpha of P-value < 0.0056 is used correcting for 9 significant variants in which heterogeneity was tested on. Variants with significant heterogeneity were excluded from this table.

Supplementary Table 6. Genetic Correlations. Bonferroni-correction was applied to account for multiple testing with 23 tests giving an adjusted alpha of P-value < 0.002. P-values are two-sided and were calculated by dividing the correlation values (rg) by their standard errors and observing the probability mass corresponding to equal or more extreme values from both tails of the standard normal distribution (as in R package glm).

Supplementary Table 7. Summary statistics used to perform genetic correlations.

Supplementary Table 8. Reference levels for logistic regression for each of the predictor categories.

Supplementary Table 9. Mendelian Randomization results exploring the causal effect of COVID-19 phenotypes, BMI, height and type 2 diabetes on COVID vaccination. P-values are two-sided and were calculated by dividing the coefficient (Beta) by their standard errors and observing the probability mass corresponding to equal or more extreme values from both tails of the standard normal distribution (as in R package glm).

FinnGen consortium full list of members

Aarno Palotie (Institute for Molecular Medicine Finland (FIMM), HiLIFE, University of Helsinki, Helsinki, Finland; Broad Institute of MIT and Harvard; Massachusetts General Hospital), Mark Daly (Institute for Molecular Medicine Finland (FIMM), HiLIFE, University of Helsinki, Helsinki, Finland; Broad Institute of MIT and Harvard; Massachusetts General Hospital), Bridget Riley-Gills (Abbvie, Chicago, IL, United States), Howard Jacob (Abbvie, Chicago, IL, United States), Dirk Paul (Astra Zeneca, Cambridge, United Kingdom), Slavé Petrovski (Astra Zeneca, Cambridge, United Kingdom), Heiko Runz (Biogen, Cambridge, MA, United States), Sally John (Biogen, Cambridge, MA, United States), George Okafo (Boehringer Ingelheim, Ingelheim am Rhein, Germany), Nathan Lawless (Boehringer Ingelheim, Ingelheim am Rhein, Germany), Heli Salminen-Mankonen (Boehringer Ingelheim, Ingelheim am Rhein, Germany), Robert Plenge (Bristol Myers Squibb, New York, NY, United States), Joseph Maranville (Bristol Myers Squibb, New York, NY, United States), Mark McCarthy (Genentech, San Francisco, CA, United States), Margaret G. Ehm (GlaxoSmithKline, Collegeville, PA, United States), Kirsi Auro (GlaxoSmithKline, Espoo, Finland), Simonne Longerich (Merck, Kenilworth, NJ, United States), Anders Mälarstig (Pfizer, New York, NY, United States), Katherine Klinger (Translational Sciences, Sanofi R&D, Framingham, MA, USA), Clement Chatelain (Translational Sciences, Sanofi R&D, Framingham, MA, USA), Matthias Gossel (Translational Sciences, Sanofi R&D, Framingham, MA, USA), Karol Estrada (Maze Therapeutics, San Francisco, CA, United States), Robert Graham (Maze Therapeutics, San Francisco, CA, United States), Robert Yang (Janssen Biotech, Beerse, Belgium), Chris O´Donnell (Novartis Institutes for BioMedical Research, Cambridge, MA, United States), Tomi P. Mäkelä (HiLIFE, University of Helsinki, Finland, Finland), Jaakko Kaprio (Institute for Molecular Medicine Finland (FIMM), HiLIFE, University of Helsinki, Helsinki, Finland), Petri Virolainen (Auria Biobank / University of Turku / Hospital District of Southwest Finland, Turku, Finland), Antti Hakanen (Auria Biobank / University of Turku / Hospital District of Southwest Finland, Turku, Finland), Terhi Kilpi (THL Biobank / Finnish Institute for Health and Welfare (THL), Helsinki, Finland), Markus Perola (THL Biobank / Finnish Institute for Health and Welfare (THL), Helsinki, Finland), Jukka Partanen (Finnish Red Cross Blood Service / Finnish Hematology Registry and Clinical Biobank, Helsinki, Finland), Anne Pitkäranta (Helsinki Biobank / Helsinki University and Hospital District of Helsinki and Uusimaa, Helsinki), Taneli Raivio (Helsinki Biobank / Helsinki University and Hospital District of Helsinki and Uusimaa, Helsinki), Jani Tikkanen (Northern Finland Biobank Borealis / University of Oulu / Northern Ostrobothnia Hospital District, Oulu, Finland), Raisa Serpi (Northern Finland Biobank Borealis / University of Oulu / Northern Ostrobothnia Hospital District, Oulu, Finland), Tarja Laitinen (Finnish Clinical Biobank Tampere / University of Tampere / Pirkanmaa Hospital District, Tampere, Finland), Veli-Matti Kosma (Biobank of Eastern Finland / University of Eastern Finland / Northern Savo Hospital District, Kuopio, Finland), Jari Laukkanen (Central Finland Biobank / University of Jyväskylä / Central Finland Health Care District, Jyväskylä, Finland), Marco Hautalahti (FINBB - Finnish biobank cooperative), Outi Tuovila (Business Finland, Helsinki, Finland), Raimo Pakkanen (Business Finland, Helsinki, Finland), Jeffrey Waring (Abbvie, Chicago, IL, United States), Bridget Riley-Gillis (Abbvie, Chicago, IL, United States), Fedik Rahimov (Abbvie, Chicago, IL, United States), Ioanna Tachmazidou (Astra Zeneca, Cambridge, United Kingdom), Chia-Yen Chen (Biogen, Cambridge, MA, United States), Zhihao Ding (Boehringer Ingelheim, Ingelheim am Rhein, Germany), Marc Jung (Boehringer

Ingelheim, Ingelheim am Rhein, Germany), Shameek Biswas (Bristol Myers Squibb, New York, NY, United States), Rion Pendergrass (Genentech, San Francisco, CA, United States), David Pulford (GlaxoSmithKline, Stevenage, United Kingdom), Neha Raghavan (Merck, Kenilworth, NJ, United States), Adriana Huertas-Vazquez (Merck, Kenilworth, NJ, United States), Jae-Hoon Sul (Merck, Kenilworth, NJ, United States), Xinli Hu (Pfizer, New York, NY, United States), Åsa Hedman (Pfizer, New York, NY, United States), Manuel Rivas (Maze Therapeutics, San Francisco, CA, United States), Dawn Waterworth (Janssen Research & Development, LLC, Spring House, PA, United States), Nicole Renaud (Novartis Institutes for BioMedical Research, Cambridge, MA, United States), Ma´en Obeidat (Novartis Institutes for BioMedical Research, Cambridge, MA, United States), Samuli Ripatti (Institute for Molecular Medicine Finland (FIMM), HiLIFE, University of Helsinki, Helsinki, Finland), Johanna Schleutker (Auria Biobank / Univ. of Turku / Hospital District of Southwest Finland, Turku, Finland), Mikko Arvas (Finnish Red Cross Blood Service / Finnish Hematology Registry and Clinical Biobank, Helsinki, Finland), Olli Carpén (Helsinki Biobank / Helsinki University and Hospital District of Helsinki and Uusimaa, Helsinki), Reetta Hinttala (Northern Finland Biobank Borealis / University of Oulu / Northern Ostrobothnia Hospital District, Oulu, Finland), Johannes Kettunen (Northern Finland Biobank Borealis / University of Oulu / Northern Ostrobothnia Hospital District, Oulu, Finland), Arto Mannermaa (Biobank of Eastern Finland / University of Eastern Finland / Northern Savo Hospital District, Kuopio, Finland), Katriina Aalto-Setälä (Faculty of Medicine and Health Technology, Tampere University, Tampere, Finland), Mika Kähönen (Finnish Clinical Biobank Tampere / University of Tampere / Pirkanmaa Hospital District, Tampere, Finland), Johanna Mäkelä (FINBB - Finnish biobank cooperative), Reetta Kälviäinen (Northern Savo Hospital District, Kuopio, Finland), Valtteri Julkunen (Northern Savo Hospital District, Kuopio, Finland), Hilkka Soininen (Northern Savo Hospital District, Kuopio, Finland), Anne Remes (Northern Ostrobothnia Hospital District, Oulu, Finland), Mikko Hiltunen (University of Eastern Finland, Kuopio, Finland), Jukka Peltola (Pirkanmaa Hospital District, Tampere, Finland), Minna Raivio (Hospital District of Helsinki and Uusimaa, Helsinki, Finland), Pentti Tienari (Hospital District of Helsinki and Uusimaa, Helsinki, Finland), Juha Rinne (Hospital District of Southwest Finland, Turku, Finland), Roosa Kallionpää (Hospital District of Southwest Finland, Turku, Finland), Juulia Partanen (Institute for Molecular Medicine Finland, HiLIFE, University of Helsinki, Finland), Ali Abbasi (Abbvie, Chicago, IL, United States), Adam Ziemann (Abbvie, Chicago, IL, United States), Nizar Smaoui (Abbvie, Chicago, IL, United States), Anne Lehtonen (Abbvie, Chicago, IL, United States), Susan Eaton (Biogen, Cambridge, MA, United States), Sanni Lahdenperä (Biogen, Cambridge, MA, United States), Natalie Bowers (Genentech, San Francisco, CA, United States), Edmond Teng (Genentech, San Francisco, CA, United States), Fanli Xu (GlaxoSmithKline, Brentford, United Kingdom), Laura Addis (GlaxoSmithKline, Brentford, United Kingdom), John Eicher (GlaxoSmithKline, Brentford, United Kingdom), Qingqin S Li (Janssen Research & Development, LLC, Titusville, NJ 08560, United States), Karen He (Janssen Research & Development, LLC, Spring House, PA, United States), Ekaterina Khramtsova (Janssen Research & Development, LLC, Spring House, PA, United States), Martti Färkkilä (Hospital District of Helsinki and Uusimaa, Helsinki, Finland), Jukka Koskela (Hospital District of Helsinki and Uusimaa, Helsinki, Finland), Sampsa Pikkarainen (Hospital District of Helsinki and Uusimaa, Helsinki, Finland), Airi Jussila (Pirkanmaa Hospital District, Tampere, Finland), Katri Kaukinen (Pirkanmaa Hospital District, Tampere, Finland), Timo Blomster (Northern Ostrobothnia Hospital District, Oulu, Finland), Mikko Kiviniemi (Northern Savo Hospital District, Kuopio, Finland), Markku Voutilainen (Hospital District of Southwest Finland, Turku, Finland), Tim Lu (Genentech, San

Francisco, CA, United States), Linda McCarthy (GlaxoSmithKline, Brentford, United Kingdom), Amy Hart (Janssen Research & Development, LLC, Spring House, PA, United States), Meijian Guan (Janssen Research & Development, LLC, Spring House, PA, United States), Jason Miller (Merck, Kenilworth, NJ, United States), Kirsi Kalpala (Pfizer, New York, NY, United States), Melissa Miller (Pfizer, New York, NY, United States), Kari Eklund (Hospital District of Helsinki and Uusimaa, Helsinki, Finland), Antti Palomäki (Hospital District of Southwest Finland, Turku, Finland), Pia Isomäki (Pirkanmaa Hospital District, Tampere, Finland), Laura Pirilä (Hospital District of Southwest Finland, Turku, Finland), Oili Kaipiainen-Seppänen (Northern Savo Hospital District, Kuopio, Finland), Johanna Huhtakangas (Northern Ostrobothnia Hospital District, Oulu, Finland), Nina Mars (Institute for Molecular Medicine Finland (FIMM), HiLIFE, University of Helsinki, Helsinki, Finland), Apinya Lertratanakul (Abbvie, Chicago, IL, United States), Coralie Viollet (AstraZeneca, Cambridge, United Kingdom), Marla Hochfeld (Bristol Myers Squibb, New York, NY, United States), Jorge Esparza Gordillo (GlaxoSmithKline, Brentford, United Kingdom), Fabiana Farias (Merck, Kenilworth, NJ, United States), Nan Bing (Pfizer, New York, NY, United States), Margit Pelkonen (Northern Savo Hospital District, Kuopio, Finland), Paula Kauppi (Hospital District of Helsinki and Uusimaa, Helsinki, Finland), Hannu Kankaanranta (University of Gothenburg, Gothenburg, Sweden/ Seinäjoki Central Hospital, Seinäjoki, Finland/ Tampere University, Tampere, Finland), Terttu Harju (Northern Ostrobothnia Hospital District, Oulu, Finland), Riitta Lahesmaa (Hospital District of Southwest Finland, Turku, Finland), Hubert Chen (Genentech, San Francisco, CA, United States), Joanna Betts (GlaxoSmithKline, Brentford, United Kingdom), Rajashree Mishra (GlaxoSmithKline, Brentford, United Kingdom), Majd Mouded (Novartis, Basel, Switzerland), Debby Ngo (Novartis, Basel, Switzerland), Teemu Niiranen (Finnish Institute for Health and Welfare (THL), Helsinki, Finland), Felix Vaura (Finnish Institute for Health and Welfare (THL), Helsinki, Finland), Veikko Salomaa (Finnish Institute for Health and Welfare (THL), Helsinki, Finland), Kaj Metsärinne (Hospital District of Southwest Finland, Turku, Finland), Jenni Aittokallio (Hospital District of Southwest Finland, Turku, Finland), Jussi Hernesniemi (Pirkanmaa Hospital District, Tampere, Finland), Daniel Gordin (Hospital District of Helsinki and Uusimaa, Helsinki, Finland), Juha Sinisalo (Hospital District of Helsinki and Uusimaa, Helsinki, Finland), Marja-Riitta Taskinen (Hospital District of Helsinki and Uusimaa, Helsinki, Finland), Tiinamaija Tuomi (Hospital District of Helsinki and Uusimaa, Helsinki, Finland), Timo Hiltunen (Hospital District of Helsinki and Uusimaa, Helsinki, Finland), Amanda Elliott (Institute for Molecular Medicine Finland (FIMM), HiLIFE, University of Helsinki, Helsinki, Finland; Broad Institute, Cambridge, MA, USA and Massachusetts General Hospital, Boston, MA, USA), Mary Pat Reeve (Institute for Molecular Medicine Finland (FIMM), HiLIFE, University of Helsinki, Helsinki, Finland), Sanni Ruotsalainen (Institute for Molecular Medicine Finland (FIMM), HiLIFE, University of Helsinki, Helsinki, Finland), Audrey Chu (GlaxoSmithKline, Brentford, United Kingdom), Dermot Reilly (Janssen Research & Development, LLC, Boston, MA, United States), Mike Mendelson (Novartis, Boston, MA, United States), Jaakko Parkkinen (Pfizer, New York, NY, United States), Tuomo Meretoja (Hospital District of Helsinki and Uusimaa, Helsinki, Finland), Heikki Joensuu (Hospital District of Helsinki and Uusimaa, Helsinki, Finland), Johanna Mattson (Hospital District of Helsinki and Uusimaa, Helsinki, Finland), Eveliina Salminen (Hospital District of Helsinki and Uusimaa, Helsinki, Finland), Annika Auranen (Pirkanmaa Hospital District , Tampere, Finland), Peeter Karihtala (Northern Ostrobothnia Hospital District, Oulu, Finland), Päivi Auvinen (Northern Savo Hospital District, Kuopio, Finland), Klaus Elenius (Hospital District of Southwest Finland, Turku, Finland), Esa Pitkänen (Institute for Molecular Medicine Finland

(FIMM), HiLIFE, University of Helsinki, Helsinki, Finland), Relja Popovic (Abbvie, Chicago, IL, United States), Margarete Fabre (AstraZeneca, Cambridge, United Kingdom), Jennifer Schutzman (Genentech, San Francisco, CA, United States), Diptee Kulkarni (GlaxoSmithKline, Brentford, United Kingdom), Alessandro Porello (Janssen Research & Development, LLC, Spring House, PA, United States), Andrey Loboda (Merck, Kenilworth, NJ, United States), Heli Lehtonen (Pfizer, New York, NY, United States), Stefan McDonough (Pfizer, New York, NY, United States), Sauli Vuoti (Janssen-Cilag Oy, Espoo, Finland), Kai Kaarniranta (Northern Savo Hospital District, Kuopio, Finland; Department of Molecular Genetics, University of Lodz, Lodz, Poland), Joni A Turunen (Helsinki University Hospital and University of Helsinki, Helsinki, Finland; Eye Genetics Group, Folkhälsan Research Center, Helsinki, Finland), Terhi Ollila (Hospital District of Helsinki and Uusimaa, Helsinki, Finland), Hannu Uusitalo (Pirkanmaa Hospital District, Tampere, Finland), Juha Karjalainen (Institute for Molecular Medicine Finland (FIMM), HiLIFE, University of Helsinki, Helsinki, Finland), Mengzhen Liu (Abbvie, Chicago, IL, United States), Stephanie Loomis (Biogen, Cambridge, MA, United States), Erich Strauss (Genentech, San Francisco, CA, United States), Hao Chen (Genentech, San Francisco, CA, United States), Kaisa Tasanen (Northern Ostrobothnia Hospital District, Oulu, Finland), Laura Huilaja (Northern Ostrobothnia Hospital District, Oulu, Finland), Katariina Hannula-Jouppi (Hospital District of Helsinki and Uusimaa, Helsinki, Finland), Teea Salmi (Pirkanmaa Hospital District, Tampere, Finland), Sirkku Peltonen (Hospital District of Southwest Finland, Turku, Finland), Leena Koulu (Hospital District of Southwest Finland, Turku, Finland), David Choy (Genentech, San Francisco, CA, United States), Ying Wu (Pfizer, New York, NY, United States), Pirkko Pussinen (Hospital District of Helsinki and Uusimaa, Helsinki, Finland), Aino Salminen (Hospital District of Helsinki and Uusimaa, Helsinki, Finland), Tuula Salo (Hospital District of Helsinki and Uusimaa, Helsinki, Finland), David Rice (Hospital District of Helsinki and Uusimaa, Helsinki, Finland), Pekka Nieminen (Hospital District of Helsinki and Uusimaa, Helsinki, Finland), Ulla Palotie (Hospital District of Helsinki and Uusimaa, Helsinki, Finland), Maria Siponen (Northern Savo Hospital District, Kuopio, Finland), Liisa Suominen (Northern Savo Hospital District, Kuopio, Finland), Päivi Mäntylä (Northern Savo Hospital District, Kuopio, Finland), Ulvi Gursoy (Hospital District of Southwest Finland, Turku, Finland), Vuokko Anttonen (Northern Ostrobothnia Hospital District, Oulu, Finland), Kirsi Sipilä (Research Unit of Oral Health Sciences Faculty of Medicine, University of Oulu, Oulu, Finland; Medical Research Center, Oulu, Oulu University Hospital and University of Oulu, Oulu, Finland), Rion Pendergrass (Genentech, San Francisco, CA, United States), Hannele Laivuori (Institute for Molecular Medicine Finland (FIMM), HiLIFE, University of Helsinki, Helsinki, Finland), Venla Kurra (Pirkanmaa Hospital District, Tampere, Finland), Laura Kotaniemi-Talonen (Pirkanmaa Hospital District, Tampere, Finland), Oskari Heikinheimo (Hospital District of Helsinki and Uusimaa, Helsinki, Finland), Ilkka Kalliala (Hospital District of Helsinki and Uusimaa, Helsinki, Finland), Lauri Aaltonen (Hospital District of Helsinki and Uusimaa, Helsinki, Finland), Varpu Jokimaa (Hospital District of Southwest Finland, Turku, Finland), Marja Vääräsmäki (Northern Ostrobothnia Hospital District, Oulu, Finland), Outi Uimari (Northern Ostrobothnia Hospital District, Oulu, Finland), Laure Morin-Papunen (Northern Ostrobothnia Hospital District, Oulu, Finland), Maarit Niinimäki (Northern Ostrobothnia Hospital District, Oulu, Finland), Terhi Piltonen (Northern Ostrobothnia Hospital District, Oulu, Finland), Katja Kivinen (Institute for Molecular Medicine Finland (FIMM), HiLIFE, University of Helsinki, Helsinki, Finland), Elisabeth Widen (Institute for Molecular Medicine Finland (FIMM), HiLIFE, University of Helsinki, Helsinki, Finland), Taru Tukiainen (Institute for Molecular Medicine Finland (FIMM), HiLIFE, University of Helsinki, Helsinki,

Finland), Niko Välimäki (University of Helsinki, Helsinki, Finland), Eija Laakkonen (University of Jyväskylä, Jyväskylä, Finland), Jaakko Tyrmi (University of Oulu, Oulu, Finland / University of Tampere, Tampere, Finland), Heidi Silven (University of Oulu, Oulu, Finland), Eeva Sliz (University of Oulu, Oulu, Finland), Riikka Arffman (University of Oulu, Oulu, Finland), Susanna Savukoski (University of Oulu, Oulu, Finland), Triin Laisk (Estonian biobank, Tartu, Estonia), Natalia Pujol (Estonian biobank, Tartu, Estonia), Janet Kumar (GlaxoSmithKline, Collegeville, PA, United States), Iiris Hovatta (University of Helsinki, Finland), Erkki Isometsä (Hospital District of Helsinki and Uusimaa, Helsinki, Finland), Hanna Ollila (Institute for Molecular Medicine Finland (FIMM), HiLIFE, University of Helsinki, Helsinki, Finland), Jaana Suvisaari (Finnish Institute for Health and Welfare (THL), Helsinki, Finland), Thomas Damm Als (Aarhus University, Denmark), Antti Mäkitie (Department of Otorhinolaryngology - Head and Neck Surgery, University of Helsinki and Helsinki University Hospital, Helsinki, Finland), Argyro Bizaki-Vallaskangas (Pirkanmaa Hospital District, Tampere, Finland), Sanna Toppila-Salmi (University of Helsinki, Finland), Tytti Willberg (Hospital District of Southwest Finland, Turku, Finland), Elmo Saarentaus (Institute for Molecular Medicine Finland (FIMM), HiLIFE, University of Helsinki, Helsinki, Finland), Antti Aarnisalo (Hospital District of Helsinki and Uusimaa, Helsinki, Finland), Elisa Rahikkala (Northern Ostrobothnia Hospital District, Oulu, Finland), Kristiina Aittomäki (Department of Medical Genetics, Helsinki University Central Hospital, Helsinki, Finland), Fredrik Åberg (Transplantation and Liver Surgery Clinic, Helsinki University Hospital, Helsinki University, Helsinki, Finland), Mitja Kurki (Institute for Molecular Medicine Finland (FIMM), HiLIFE, University of Helsinki, Helsinki, Finland; Broad Institute, Cambridge, MA, United States), Aki Havulinna (Institute for Molecular Medicine Finland (FIMM), HiLIFE, University of Helsinki, Helsinki, Finland; Finnish Institute for Health and Welfare (THL), Helsinki, Finland), Juha Mehtonen (Institute for Molecular Medicine Finland (FIMM), HiLIFE, University of Helsinki, Helsinki, Finland), Priit Palta (Institute for Molecular Medicine Finland (FIMM), HiLIFE, University of Helsinki, Helsinki, Finland), Shabbeer Hassan (Institute for Molecular Medicine Finland (FIMM), HiLIFE, University of Helsinki, Helsinki, Finland), Pietro Della Briotta Parolo (Institute for Molecular Medicine Finland (FIMM), HiLIFE, University of Helsinki, Helsinki, Finland), Wei Zhou (Broad Institute, Cambridge, MA, United States), Mutaamba Maasha (Broad Institute, Cambridge, MA, United States), Susanna Lemmelä (Institute for Molecular Medicine Finland (FIMM), HiLIFE, University of Helsinki, Helsinki, Finland), Aoxing Liu (Institute for Molecular Medicine Finland (FIMM), HiLIFE, University of Helsinki, Helsinki, Finland), Arto Lehisto (Institute for Molecular Medicine Finland (FIMM), HiLIFE, University of Helsinki, Helsinki, Finland), Andrea Ganna (Institute for Molecular Medicine Finland (FIMM), HiLIFE, University of Helsinki, Helsinki, Finland), Vincent Llorens (Institute for Molecular Medicine Finland (FIMM), HiLIFE, University of Helsinki, Helsinki, Finland), Henrike Heyne (Institute for Molecular Medicine Finland (FIMM), HiLIFE, University of Helsinki, Helsinki, Finland), Joel Rämö (Institute for Molecular Medicine Finland (FIMM), HiLIFE, University of Helsinki, Helsinki, Finland), Rodos Rodosthenous (Institute for Molecular Medicine Finland (FIMM), HiLIFE, University of Helsinki, Helsinki, Finland), Satu Strausz (Institute for Molecular Medicine Finland (FIMM), HiLIFE, University of Helsinki, Helsinki, Finland), Tuula Palotie (University of Helsinki and Hospital District of Helsinki and Uusimaa, Helsinki, Finland), Kimmo Palin (University of Helsinki, Helsinki, Finland), Javier Garcia-Tabuenca (University of Tampere, Tampere, Finland), Harri Siirtola (University of Tampere, Tampere, Finland), Tuomo Kiiskinen (Institute for Molecular Medicine Finland (FIMM), HiLIFE, University of Helsinki, Helsinki, Finland), Jiwoo Lee (Institute for Molecular Medicine Finland (FIMM), HiLIFE, University of Helsinki, Helsinki, Finland; Broad Institute, Cambridge, MA,

United States), Kristin Tsuo (Institute for Molecular Medicine Finland (FIMM), HiLIFE, University of Helsinki, Helsinki, Finland; Broad Institute, Cambridge, MA, United States), Kati Kristiansson (THL Biobank / Finnish Institute for Health and Welfare (THL), Helsinki, Finland), Kati Hyvärinen (Finnish Red Cross Blood Service, Helsinki, Finland), Jarmo Ritari (Finnish Red Cross Blood Service, Helsinki, Finland), Katri Pylkäs (University of Oulu, Oulu, Finland), Minna Karjalainen (University of Oulu, Oulu, Finland), Tuomo Mantere (Northern Finland Biobank Borealis / University of Oulu / Northern Ostrobothnia Hospital District, Oulu, Finland), Eeva Kangasniemi (Finnish Clinical Biobank Tampere / University of Tampere / Pirkanmaa Hospital District, Tampere, Finland), Sami Heikkinen (University of Eastern Finland, Kuopio, Finland), Nina Pitkänen (Auria Biobank / University of Turku / Hospital District of Southwest Finland, Turku, Finland), Samuel Lessard (Translational Sciences, Sanofi R&D, Framingham, MA, USA), Clément Chatelain (Translational Sciences, Sanofi R&D, Framingham, MA, USA), Lila Kallio (Auria Biobank / University of Turku / Hospital District of Southwest Finland, Turku, Finland), Tiina Wahlfors (THL Biobank / Finnish Institute for Health and Welfare (THL), Helsinki, Finland), Eero Punkka (Helsinki Biobank / Helsinki University and Hospital District of Helsinki and Uusimaa, Helsinki), Sanna Siltanen (Finnish Clinical Biobank Tampere / University of Tampere / Pirkanmaa Hospital District, Tampere, Finland), Teijo Kuopio (Central Finland Biobank / University of Jyväskylä / Central Finland Health Care District, Jyväskylä, Finland), Anu Jalanko (Institute for Molecular Medicine Finland (FIMM), HiLIFE, University of Helsinki, Helsinki, Finland), Huei-Yi Shen (Institute for Molecular Medicine Finland (FIMM), HiLIFE, University of Helsinki, Helsinki, Finland), Risto Kajanne (Institute for Molecular Medicine Finland (FIMM), HiLIFE, University of Helsinki, Helsinki, Finland), Mervi Aavikko (Institute for Molecular Medicine Finland (FIMM), HiLIFE, University of Helsinki, Helsinki, Finland), Rasko Leinonen (Institute for Molecular Medicine Finland (FIMM), HiLIFE, University of Helsinki, Helsinki, Finland; European Molecular Biology Laboratory, European Bioinformatics Institute, Cambridge, UK), Henna Palin (Finnish Clinical Biobank Tampere / University of Tampere / Pirkanmaa Hospital District, Tampere, Finland), Malla-Maria Linna (Helsinki Biobank / Helsinki University and Hospital District of Helsinki and Uusimaa, Helsinki), Masahiro Kanai (Broad Institute, Cambridge, MA, United States), Zhili Zheng (Broad Institute, Cambridge, MA, United States), L. Elisa Lahtela (Institute for Molecular Medicine Finland (FIMM), HiLIFE, University of Helsinki, Helsinki, Finland), Mari Kaunisto (Institute for Molecular Medicine Finland (FIMM), HiLIFE, University of Helsinki, Helsinki, Finland), Elina Kilpeläinen (Institute for Molecular Medicine Finland (FIMM), HiLIFE, University of Helsinki, Helsinki, Finland), Timo P. Sipilä (Institute for Molecular Medicine Finland (FIMM), HiLIFE, University of Helsinki, Helsinki, Finland), Oluwaseun Alexander Dada (Institute for Molecular Medicine Finland (FIMM), HiLIFE, University of Helsinki, Helsinki, Finland), Awaisa Ghazal (Institute for Molecular Medicine Finland (FIMM), HiLIFE, University of Helsinki, Helsinki, Finland), Anastasia Kytölä (Institute for Molecular Medicine Finland (FIMM), HiLIFE, University of Helsinki, Helsinki, Finland), Rigbe Weldatsadik (Institute for Molecular Medicine Finland (FIMM), HiLIFE, University of Helsinki, Helsinki, Finland), Kati Donner (Institute for Molecular Medicine Finland (FIMM), HiLIFE, University of Helsinki, Helsinki, Finland), Anu Loukola (Helsinki Biobank / Helsinki University and Hospital District of Helsinki and Uusimaa, Helsinki), Päivi Laiho (THL Biobank / Finnish Institute for Health and Welfare (THL), Helsinki, Finland), Tuuli Sistonen (THL Biobank / Finnish Institute for Health and Welfare (THL), Helsinki, Finland), Essi Kaiharju (THL Biobank / Finnish Institute for Health and Welfare (THL), Helsinki, Finland), Markku Laukkanen (THL Biobank / Finnish Institute for Health and Welfare (THL), Helsinki, Finland), Elina Järvensivu (THL Biobank / Finnish Institute for Health and Welfare (THL), Helsinki,

Finland), Sini Lähteenmäki (THL Biobank / Finnish Institute for Health and Welfare (THL), Helsinki, Finland), Lotta Männikkö (THL Biobank / Finnish Institute for Health and Welfare (THL), Helsinki, Finland), Regis Wong (THL Biobank / Finnish Institute for Health and Welfare (THL), Helsinki, Finland), Auli Toivola (THL Biobank / Finnish Institute for Health and Welfare (THL), Helsinki, Finland), Minna Brunfeldt (THL Biobank / Finnish Institute for Health and Welfare (THL), Helsinki, Finland), Hannele Mattsson (THL Biobank / Finnish Institute for Health and Welfare (THL), Helsinki, Finland), Sami Koskelainen (THL Biobank / Finnish Institute for Health and Welfare (THL), Helsinki, Finland), Tero Hiekkalinna (THL Biobank / Finnish Institute for Health and Welfare (THL), Helsinki, Finland), Teemu Paajanen (THL Biobank / Finnish Institute for Health and Welfare (THL), Helsinki, Finland), Kalle Pärn (Institute for Molecular Medicine Finland (FIMM), HiLIFE, University of Helsinki, Helsinki, Finland), Mart Kals (Institute for Molecular Medicine Finland (FIMM), HiLIFE, University of Helsinki, Helsinki, Finland), Shuang Luo (Institute for Molecular Medicine Finland (FIMM), HiLIFE, University of Helsinki, Helsinki, Finland), Shanmukha Sampath Padmanabhuni (Institute for Molecular Medicine Finland (FIMM), HiLIFE, University of Helsinki, Helsinki, Finland), Marianna Niemi (University of Tampere, Tampere, Finland), Javier Gracia-Tabuenca (University of Tampere, Tampere, Finland), Mika Helminen (University of Tampere, Tampere, Finland), Tiina Luukkaala (University of Tampere, Tampere, Finland), Iida Vähätalo (University of Tampere, Tampere, Finland), Jyrki Tammerluoto (Institute for Molecular Medicine Finland (FIMM), HiLIFE, University of Helsinki, Helsinki, Finland), Sarah Smith (Finnish Biobank Cooperative - FINBB), Tom Southerington (Finnish Biobank Cooperative - FINBB), Petri Lehto (Finnish Biobank Cooperative - FINBB)
