# Supplementary material for: Nationwide health, socio-economic and genetic predictors of COVID-19 vaccination status in Finland
Source: Nat Hum Behav. 2023 Apr 20;7(7):1069–83. doi: 10.1038/s41562-023-01591-z (PMC10365990; doi:10.1038/s41562-023-01591-z)
Supplement: Supplementary file 2 — Reporting Summary [file 41562_2023_1591_MOESM2_ESM.pdf]

nature portfolio

Corresponding author(s): Andrea Ganna

Last updated by author(s): Mar 8, 2023

Reporting Summary

Nature Portfolio wishes to improve the reproducibility of the work that we publish. This form provides structure for consistency and transparency in reporting. For further information on Nature Portfolio policies, see our Editorial Policies and the Editorial Policy Checklist.

Statistics

For all statistical analyses, confirm that the following items are present in the figure legend, table legend, main text, or Methods section.

n/a	Confirmed	
☐	☒	The exact sample size (*n*) for each experimental group/condition, given as a discrete number and unit of measurement
☐	☒	A statement on whether measurements were taken from distinct samples or whether the same sample was measured repeatedly
☐	☒	The statistical test(s) used AND whether they are one- or two-sided<br>*Only common tests should be described solely by name; describe more complex techniques in the Methods section.*
☐	☒	A description of all covariates tested
☐	☒	A description of any assumptions or corrections, such as tests of normality and adjustment for multiple comparisons
☐	☒	A full description of the statistical parameters including central tendency (e.g. means) or other basic estimates (e.g. regression coefficient) AND variation (e.g. standard deviation) or associated estimates of uncertainty (e.g. confidence intervals)
☐	☒	For null hypothesis testing, the test statistic (e.g. *F*, *t*, *r*) with confidence intervals, effect sizes, degrees of freedom and *P* value noted<br>*Give P values as exact values whenever suitable.*
☒	☐	For Bayesian analysis, information on the choice of priors and Markov chain Monte Carlo settings
☒	☐	For hierarchical and complex designs, identification of the appropriate level for tests and full reporting of outcomes
☐	☒	Estimates of effect sizes (e.g. Cohen's *d*, Pearson's *r*), indicating how they were calculated

Our web collection on statistics for biologists contains articles on many of the points above.

Software and code

Policy information about availability of computer code

Data collection	No software was used for data collection.
Data analysis	All data analysis steps are described in Methods. Briefly, logistic regression analyses were performed with R using bigglm from library biglm (version 0.9.2.1), and multiple hypothesis testing corrected p-values were computed using Python package statsmodels (version 0.12.2). XGBoost analyses were performed with the Python library xgboost (version 1.5.0), with hyperparameters optimized using Bayesian hyperparameter optimization (BayesSearchCV function from scikit-optimize, version 0.9.0). XGBoost model feature importances were computed using the shap software (version 0.39.0). Lasso classifiers were fitted with the cv.glmnet function from the glmnet R package (version 4.1.1). Partial correlations were computed using partial_corr function from Python library pingouin (version 0.5.2). Clustering of the partial correlation coefficient matrix was computed and the heatmap plotted using the clustermap function from Python library seaborn (version 0.11.2). GWAS was performed using REGENIE v2.2.4 for FinnGen and SAIGE v1.0.7 for Estonian Biobank. Genetic correlations were computed using Linkage Disequilibrium Score Regression (LDSC, version 1.0.1). Meta-analysis was performed using METAL (version released on 2011-03-25). Polygenic Scores (PGS) were computed using PRS-CS (version released on 2022-11-03). MRBase (TwoSampleMR, version 0.5.6) was used to run two sample mendelian randomization. The most likely gene linked to each lead variant in the GWAS was reported based on a machine learning-based prioritization score from Open Targets Genetics. The essential custom code developed for this study is available in Github at: https://github.com/dsgelab/COVID-19-vaccination-public.

For manuscripts utilizing custom algorithms or software that are central to the research but not yet described in published literature, software must be made available to editors and reviewers. We strongly encourage code deposition in a community repository (e.g. GitHub). See the Nature Portfolio guidelines for submitting code & software for further information.

Data

Policy information about availability of data

All manuscripts must include a data availability statement. This statement should provide the following information, where applicable:

- Accession codes, unique identifiers, or web links for publicly available datasets
- A description of any restrictions on data availability
- For clinical datasets or third party data, please ensure that the statement adheres to our policy

Data dictionaries for FinRegistry are publicly available on the FinRegistry website (www.finregistry.fi/finnish-registry-data). Access to FinRegistry data can be obtained by submitting a data permit application for individual-level data for the Finnish social and health data permit authority Findata (https://asiointi.findata.fi/). The application includes information on the purpose of data use; the requested data, including the variables, definitions for the target and control groups, and external datasets to be combined with FinRegistry data; the dates of the data needed; and a data utilization plan. The requests are evaluated on a case-by-case basis. Once approved, the data are sent to a secure computing environment Kapseli and can be accessed within the European Economic Area (EEA) and within countries with an adequacy decision from the European Commission.

The Finnish biobank data can be accessed through the Fingenious® services (https://site.fingenious.fi/en/) managed by FINBB.

Access to Estonian biobank data (https://genomics.ut.ee/en/content/estonian-biobank) is restricted for approved researchers and can be requested.

Summary statistics of the COVID-19 vaccination uptake GWAS are available at the GWAS catalog with accession code GCP000553.

Human research participants

Policy information about studies involving human research participants and Sex and Gender in Research.

Reporting on sex and gender	Sex (as recorded in the nation-wide registers) was used as a covariate in all of the presented analyses.
Population characteristics	The FinRegistry dataset includes all individuals alive and living in Finland on 31.10.2021, aged between 30-80. Individuals having received a laboratory confirmed COVID-19 diagnosis prior to 31.10.2021, or who lived in one municipality with insufficient vaccination records were further excluded. After these exclusion criteria, the final FinRegistry study population contains 3,192,505 individuals. The FinnGen study population includes 273,615 of these individuals with genetic information measured. Similar inclusion and exclusion criteria were applied to the Estonian biobank, resulting in 202,910 individuals with genetic information measured.
Recruitment	The FinRegistry dataset includes each individual alive and living in Finland on 1.1.2010.
Ethics oversight	FinRegistry is a collaboration project of the Finnish Institute for Health and Welfare (THL) and the Data Science Genetic Epidemiology research group at the Institute for Molecular Medicine Finland (FIMM), University of Helsinki. The FinRegistry project has received the following approvals for data access from the National Institute of Health and Welfare (THL/1776/6.02.00/2019 and subsequent amendments), DVV (VRK/5722/2019-2), Finnish Center for Pension (ETK/SUTI 22003) and Statistics Finland (TK-53-1451-19). The FinRegistry project has received IRB approval from the National Institute of Health and Welfare (Kokous 7/2019).<br><br>Patients and control subjects in FinnGen provided informed consent for biobank research, based on the Finnish Biobank Act. Alternatively, separate research cohorts, collected prior the Finnish Biobank Act came into effect (in September 2013) and start of FinnGen (August 2017), were collected based on study-specific consents and later transferred to the Finnish biobanks after approval by Fimea (Finnish Medicines Agency), the National Supervisory Authority for Welfare and Health. Recruitment protocols followed the biobank protocols approved by Fimea. The Coordinating Ethics Committee of the Hospital District of Helsinki and Uusimaa (HUS) statement number for the FinnGen study is Nr HUS/990/2017.<br>The FinnGen study is approved by Finnish Institute for Health and Welfare (permit numbers: THL/2031/6.02.00/2017, THL/1101/5.05.00/2017, THL/341/6.02.00/2018, THL/2222/6.02.00/2018, THL/283/6.02.00/2019, THL/1721/5.05.00/2019 and THL/1524/5.05.00/2020), Digital and population data service agency (permit numbers: VRK43431/2017-3, VRK/6909/2018-3, VRK/4415/2019-3), the Social Insurance Institution (permit numbers: KELA 58/522/2017, KELA 131/522/2018, KELA 70/522/2019, KELA 98/522/2019, KELA 134/522/2019, KELA 138/522/2019, KELA 2/522/2020, KELA 16/522/2020), Findata permit numbers THL/2364/14.02/2020, THL/4055/14.06.00/2020,,THL/3433/14.06.00/2020, THL/4432/14.06/2020, THL/5189/14.06/2020, THL/5894/14.06.00/2020, THL/6619/14.06.00/2020, THL/209/14.06.00/2021, THL/688/14.06.00/2021, THL/1284/14.06.00/2021, THL/1965/14.06.00/2021, THL/5546/14.02.00/2020, THL/2658/14.06.00/2021, THL/4235/14.06.00/202, Statistics Finland (permit numbers: TK-53-1041-17 and TK/143/07.03.00/2020 (earlier TK-53-90-20) TK/1735/07.03.00/2021, TK/3112/07.03.00/2021) and Finnish Registry for Kidney Diseases permission/extract from the meeting minutes on 4th July 2019.<br>The Biobank Access Decisions for FinnGen samples and data utilized in FinnGen Data Freeze 9 include: THL Biobank BB2017_55, BB2017_111, BB2018_19, BB_2018_34, BB_2018_67, BB2018_71, BB2019_7, BB2019_8, BB2019_26, BB2020_1, Finnish Red Cross Blood Service Biobank 7.12.2017, Helsinki Biobank HUS/359/2017, HUS/248/2020, Auria Biobank AB17-5154 and amendment #1 (August 17 2020), AB20-5926 and amendment #1 (April 23 2020) and it´s modification (Sep 22 2021), Biobank Borealis of Northern Finland_2017_1013, Biobank of Eastern Finland 1186/2018 and amendment 22 § /2020, Finnish Clinical Biobank Tampere MH0004 and amendments (21.02.2020 & 06.10.2020), Central Finland Biobank 1-2017, and Terveystalo Biobank STB 2018001 and amendment 25th Aug 2020.<br><br>The activities of the Estonian Biobank (EstBB) are regulated by the Human Genes Research Act, which was adopted in 2000

specifically for the operations of the EstBB. Analysis of individual level data from the EstBB was carried out under ethical approval 1.1-12/3022 from the Estonian Committee on Bioethics and Human Research (Estonian Ministry of Social Affairs), and according to data release application 3-10/GI/31487 from the Estonian Biobank, Institute of Genomics, University of Tartu.

Note that full information on the approval of the study protocol must also be provided in the manuscript.

Field-specific reporting

Please select the one below that is the best fit for your research. If you are not sure, read the appropriate sections before making your selection.

☒ Life sciences ☐ Behavioural & social sciences ☐ Ecological, evolutionary & environmental sciences

For a reference copy of the document with all sections, see nature.com/documents/nr-reporting-summary-flat.pdf

Life sciences study design

All studies must disclose on these points even when the disclosure is negative.

Sample size

The FinRegistry dataset includes each individual alive and living in Finland on 1.1.2010, which means the entire population of Finland aged 30-80 on 31.10.2021 is included in the study. From FinnGen and Estonia biobank, all genotyped individuals meeting the inclusion criteria were selected.

Data exclusions

To restrict the study population to individuals who had had a fair opportunity of receiving the first dose of a COVID-19 vaccination by the end of October 2021, we excluded the following individuals:

Individuals who had died or emigrated before 31.12.2020 (death statistics for year 2021 in Finland were not available).
Individuals who were less than 30 years old at 31.10.2021.
Individuals who were older than 80 years old at 31.10.2021.
Individuals who had a laboratory-confirmed COVID-19 diagnosis prior to 31.10.2021.
Individuals living in a municipality called Askola.

For the genetic analyses conducted in FinnGen and Estonian Biobank, death or emigration was limited to 31.12.2019 as statistics beyond this date were unavailable for FinnGen. Residents of Askola were excluded, as it was the only municipality where the vaccination coverage differed radically from any other Finnish municipality.

Replication

The GWAS of vaccination uptake was first performed in the FinnGen sample, and replicating the analysis in one independent cohort, the Estonian biobank. The genetic correlation between these analysis was high (0.8, 95% CI: 0.66-0.95).

Randomization

Not relevant, as division to cases and controls was made according to the vaccination status and a nationwide sample was used.

Blinding

Analyses were performed using computational algorithms for large data sets of registry and genetic data and blinding was thus not relevant for this study.

Reporting for specific materials, systems and methods

We require information from authors about some types of materials, experimental systems and methods used in many studies. Here, indicate whether each material, system or method listed is relevant to your study. If you are not sure if a list item applies to your research, read the appropriate section before selecting a response.

Materials & experimental systems

n/a	Involved in the study	
☒	☐	Antibodies
☒	☐	Eukaryotic cell lines
☒	☐	Palaeontology and archaeology
☒	☐	Animals and other organisms
☒	☐	Clinical data
☒	☐	Dual use research of concern

Methods

n/a	Involved in the study	
☒	☐	ChIP-seq
☒	☐	Flow cytometry
☒	☐	MRI-based neuroimaging
